# Mosquitoes Reared in Nearby Insectaries at the Same Institution Have Significantly Divergent Microbiomes

**DOI:** 10.1111/1462-2920.70027

**Published:** 2025-01-08

**Authors:** Laura E. Brettell, Ananya F. Hoque, Tara S. Joseph, Vishaal Dhokiya, Emily A. Hornett, Grant L. Hughes, Eva Heinz

**Affiliations:** ^1^ Department of Vector Biology Liverpool School of Tropical Medicine Liverpool UK; ^2^ School of Science, Engineering and Environment University of Salford Manchester UK; ^3^ The Roslin Institute, Royal (Dick) School of Veterinary Studies The University of Edinburgh Midlothian UK; ^4^ Department of Tropical Disease Biology, Centre for Neglected Tropical Diseases Liverpool School of Tropical Medicine Liverpool UK; ^5^ Department of Evolution, Ecology and Behaviour University of Liverpool Liverpool UK; ^6^ Department of Clinical Sciences Liverpool School of Tropical Medicine Liverpool UK; ^7^ Strathclyde Institute of Pharmacy and Biomedical Sciences University of Strathclyde Glasgow UK

**Keywords:** *Aedes*, development, diversity, environment, humidity, microbiome, temperature

## Abstract

The microbiome influences critical aspects of mosquito biology and variations in microbial composition can impact the outcomes of laboratory studies. To investigate how biotic and abiotic conditions in an insectary affect the composition of the mosquito microbiome, a single cohort of *Aedes aegypti* eggs was divided into three batches and transferred to three different climate‐controlled insectaries within the Liverpool School of Tropical Medicine. The bacterial microbiome composition was compared as mosquitoes developed, the microbiome of the mosquitoes' food sources was characterised, environmental conditions over time in each insectary were measured, and mosquito development and survival were recorded. While developmental success was similar across all three insectaries, differences in microbiome composition were observed between mosquitoes from each insectary. Environmental conditions and bacterial input via food sources varied between insectaries, potentially contributing to the observed differences in microbiome composition. At both adult and larval stages, specific members of the mosquito microbiome were associated with particular insectaries; the insectary with less stable and cooler conditions resulted in a slower pupation rate and higher diversity of the larval microbiome. These findings underscore that even minor inconsistencies in rearing conditions can affect the composition of the mosquito microbiome, which may influence experimental outcomes.

## Graphical abstract

To assess the impact of minor variations in biotic and abiotic conditions within insectaries on mosquito microbiomes, we divided a cohort of eggs and reared them in three distinct insectaries. We then compared the microbiomes of the food and environment, rearing conditions (temperature, humidity), as well as mosquito development times and microbiomes throughout their development.

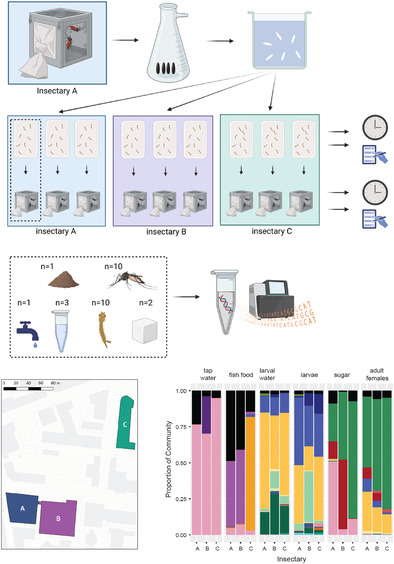

## 1 Introduction

The microbiome profoundly affects diverse aspects of mosquito biology. It is critical for larval development and influences survival, reproduction and immunity (Cansado‐Utrilla et al. 2021; Martinson and Strand 2021; Salgado et al. 2024). The microbiome can furthermore impact the transmission of pathogens by mosquitoes; either indirectly by impacting mosquito life span or reproduction, or directly by interfering with or facilitating pathogen establishment in the host (Cansado‐Utrilla et al. 2021; Hughes et al. 2014). Indeed, microbial‐based control strategies are proving to be successful avenues for vector control (Ross et al. 2022). However, our understanding of both how the microbiome affects the mosquito host, and how its assembly as a complex community takes place, is far from complete.

The composition of the mosquito microbiome can vary substantially depending on a range of biotic and abiotic factors. The microbiomes of field‐caught mosquitoes are affected by host species, geography and local climate (Bascuñán et al. 2018; Hegde et al. 2018; Jeffries et al. 2024; Medeiros et al. 2021). Laboratory‐reared mosquitoes commonly used for experimental studies, on the other hand, harbour a simpler microbiome, and mosquitoes respond differently to these microbiomes of differing complexities (Hegde et al. 2024; Santos et al. 2023). It has become apparent that despite the relative stability of the insectary environment, microbiome differences can be seen between both species and between genetically homogenous and inbred mosquito lines (i.e., the same species derived from different field‐collected individuals) under the same rearing conditions (Coon et al. 2014; Kozlova et al. 2021; Saab et al. 2020).

Laboratory studies using *Aedes aegypti* , the major vector of arboviruses including dengue, Zika and yellow fever viruses, have shown variations in the microbiome between generations and when transferred to new institutions (Accoti et al. 2023; Saab et al. 2020). Conversely, another study found mosquitoes from diverse geographic origins reared in a common insectary environment harboured remarkably similar microbiomes (Dickson et al. 2018). Taken together, these results strongly suggest the local insectary environment or rearing conditions affect microbiome composition. This perhaps is unsurprising, since bacteria are readily taken up by mosquitoes through feeding as larvae and adults (Coon, Hegde, and Hughes 2022; Kulkarni et al. 2021; MacLeod, Dimopoulos, and Short 2021). However, other studies have reported different *Ae. aegypti* lines, reared in the same insectary environment, show differences in their microbiome composition demonstrating the role of the host in microbiome selection (Kozlova et al. 2021; Short et al. 2017).

Given the complex reciprocal interactions, it can be challenging to disentangle the role of the host, the environment (e.g., larval water) and abiotic conditions (e.g., temperature) on host‐associated microbiome composition. In human disease research, a ‘reproducibility crisis’ has implicated the gut microbiome as a critical determinant of the reproducibility and translatability of research performed using animal models (Dirnagl, Bannach‐Brown, and McCann 2022). In particular, work with laboratory‐reared mice with the same genetic background has found strong facility effects on the microbiome (Parker et al. 2018). This has resulted in researchers recommending the reporting or consideration of microbiome composition in studies using laboratory mice (Ericsson and Franklin 2021). Similarly, elucidating these interactions in mosquitoes has implications for interpreting results of laboratory‐based studies, in particular considering the impact the microbiome can have on pathogen transmission, which has notoriously been variable (Bennett et al. 2002; Gubler and Rosen 1976; Kilpatrick et al. 2010; Roundy et al. 2017; Tesh, Gubler, and Rosen 1976).

To understand the influence of the insectary environment on the mosquito microbiome without the confounding effects of host genetics and potential vertically transmitted microbiome components, we reared mosquitoes from a single cohort of *Ae. aegypti* eggs in three different insectaries and characterised their bacterial microbiome composition at both the larval and adult life stages. Complementary to this, we assessed the microbiome composition of input food sources used for rearing, recorded environmental conditions within the insectaries, and noted host development times and survival rates. Our work furthers the understanding of the relative influence that host and environment exert on the microbiome composition in mosquitoes. We conclude that it is important to understand and characterise the mosquito microbiome for the accurate evaluation of laboratory studies using mosquitoes.

## 2 Materials and Methods

### 2.1 Experimental Setup

**FIGURE 1:**
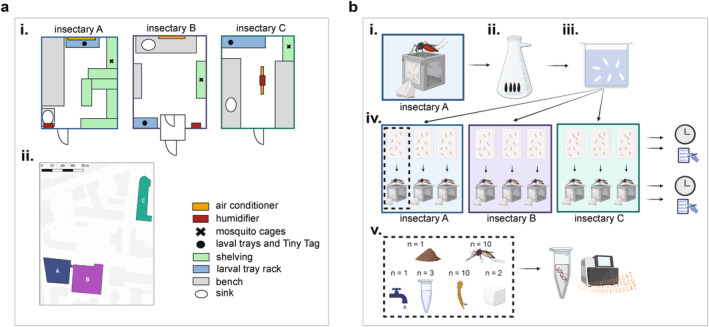
Layout of the insectaries used in this experiment and experimental setup. (a) Schematic showing the layouts of each individual insectary used in this experiment, with (i) placement locations of mosquito trays and cages and (ii) map showing locations of the three buildings where insectaries are located. (b) Experimental setup. (i) Conventionally reared *Ae. aegytpi* (Liverpool line) that had been continually reared in ‘Insectary A’ at the Liverpool School of Tropical Medicine (LSTM) were allowed to lay eggs under standard conditions. (ii) One cohort of eggs was vacuum‐hatched in the laboratory. (iii) The resulting L1 larvae were divided into nine trays of 150 larvae. (iv) Three replicate trays were transferred into each of three insectaries at LSTM: The original insectary ‘Insectary A’, and two further insectaries ‘Insectary B’ and ‘Insectary C’. Here, the cohorts were reared to adulthood according to standard conditions, recording the number of individuals that successfully developed to pupal and adult life stages. Recordings were always made between 09:00 and 12:00. TinyTag data loggers were used to measure the temperature and humidity throughout the experiment. (v) For each of the three replicates in each of the three insectaries (shown in the dashed line box), the following samples were collected: 1 fish food sample, 1 tap water sample, 3 larval water samples and 10 L3/L4 larvae samples collected at the same time, 2 sugar solution samples and 10 adult females. One additional tap water sample was also collected from each insectary. Samples were then stored at −80°C, before (vi) DNA extraction along with an additional extraction blank per batch and 16S rRNA sequencing. Panel (a ii) was created with QGIS version: Version 3.28, https://www.gqis.org/ Basemap: Positron, Map tiles by CartoDB, under CC BY 3.0. Data by OpenStreetMap, under ODbL. Panel (b) was created with Biorender.com.

The study took place across three different insectaries (here called A, B and C), within 200 m of each other at the Liverpool School of Tropical Medicine (LSTM) (Figure 1a). All insectaries are regularly used by multiple research groups to maintain long‐term mosquito lines and to carry out mosquito experiments. During the experiment, Insectary A also housed colonies of *Anopheles gambiae* , *Anopheles stephensi*, *Aedes albopictus* and additional *Ae. aegypti* lines. Insectary B housed a colony of *Culex pipiens* and there were no other mosquitoes in Insectary C. The insectaries resource fish food from the same provider. The three insectaries' conditions were set according to standard user protocols of 27°C/75% relative humidity (RH) (Insectary A), 25°C/60% RH (Insectary B) and 26°C/75% RH (Insectary C) (Table S1). The three insectaries were set at different conditions to allow for a favourable environment for the specific mosquito species housed there, with Insectary B being commonly used to rear temperate mosquito species and Insectaries A and C for tropical/subtropical species. To monitor temperature (°C) and relative humidity (%), a Tinytag Ultra 2 data logger (Gemini data loggers, UK) was placed within each insectary next to larval trays, recording every 15 min for the duration of the experiment. While the insectaries are within the same institution, they are in buildings differing in age; 2007 (Insectary A), 1903/1904 and refurbished 2010/2012 (Insectary B) and 2017 (Insectary C). The water supply for all buildings originates from the same supply but is distributed separately for each building from different cisterns and pipework.

A cohort of eggs was derived from a single colony of *Ae. aegypti* reared in Insectary A (Figure 1b). The mosquitoes belonged to the ‘Liverpool line’ that are descendants of an original West African colony brought into the laboratory in 1936 and which are continually maintained at LSTM (Ramachandran, Edeson, and Kershaw 1960). The colony used to generate eggs for this study included 300–400 adult females which were provided with fresh human blood from the National Health Service before being provided with moist filter paper to lay eggs. The resulting egg paper was dried before splitting into small segments which were randomly assigned to three equal batches. These segments were vacuum hatched for 45 min in tap water sourced from each respective insectary. The hatched larvae were then transferred to the three insectaries, fed with one spoon (approximately 0.3 g) of TetraMin fish food (Tetra), and placed in a larval tray with 1 L tap water overnight to develop. The tap water and fish food were obtained from each insectary's own taps/stocks, with the fish food from Insectaries A and B originating from the same batch and the fish food from Insectary C from a different batch. Trays were cleaned between uses with hot soapy water and were kept in each insectary, with Insectaries A and B routinely sharing trays. Four replicate samples of tap water (2 mL per sample) and three fish food (0.3 g per sample) were collected per insectary for microbiome analysis and stored at −80°C. The following day, in each insectary larvae were further split into three new replicate trays with 150 larvae per tray. Each tray was fed with 0.3 g of fish food every 2 days and monitored daily for survival. Pupation began on Day 7, at which point pupae from each tray were picked and transferred to a small container of fresh tap water within a corresponding cage. Pupae were picked for 3 days in total between 09:00–12:00, after which the number of larvae which had failed to develop were recorded. Each cage of adults was provided with a sugar solution (10% sucrose) throughout the experiment. The sugar solution is routinely prepared by combining table sugar with distilled water in a glass bottle that has been cleaned with hot soapy water. Distilled water was obtained from the nearest available source, which is the same for Insectaries A and B and different for Insectary C. Stocks of sugar solution were stored on a benchtop in each insectary, and replenished once empty and samples for sequencing were collected before providing to mosquitoes. Ten individual larvae were collected from each tray when they reached the L3/L4 stage, along with three replicate samples of larval water per tray (2 mL per tray). Numbers of hatched adults were counted on Day 14. Ten adult females were collected from each cage at 3–5 days post‐emergence (Days 12–14) and two replicate sugar solution samples (2 mL per sample) were collected per cage. Larvae and adult mosquitos were surface sterilised in 70% ethanol, then washed and stored in sterile 1 × PBS. All samples were frozen at −80°C until processed.

### 2.2 DNA Extraction and Library Preparation

Genomic DNA from all samples was extracted using a Qiagen DNA Blood and Tissue kit with modified protocols. For insect tissue (whole adults and larvae), samples were homogenised in sterile 1 × phosphate‐buffered saline (PBS) and incubated with 80 μL proteinase K and 180 μL ATL lysis buffer for 3 h at 56°C. The remaining extraction steps were performed following the manufacturer's supplementary protocol for DNA extraction from insect cells. Water (both tap water and larval water) and sugar solution samples (10% sucrose) were first centrifuged at 8000 rpm for 10 min. Then, the supernatant was removed and pellets were resuspended in 180 μL enzymatic lysis buffer (containing 20 mM Tris‐Cl (pH 8.0), 2 mM sodium EDTA, 1.2% Triton X‐100 and 20 mg/mL lysozyme) and incubated for 30 min at 37°C. Samples were then incubated with 25 μL proteinase K and 200 μL buffer AL at 56°C for 30 min, before continuing the subsequent steps from the manufacturer's instructions. For fish food samples, 2 mL sterile 1 ×  PBS was added to each 0.3 g sample and vortexed to obtain a homogenous mixture. Samples were then centrifuged at 8000 rpm for 10 min and the pellet was subjected to DNA extraction following the above protocols. A blank extraction control (extraction process used for water and sugar solution samples, but with sterile water as input) was included with each batch of DNA extractions (*n* = 7) to account for extraction or kit contaminants.

DNA was quantified using fluorometry (Qubit) and shipped on dry ice to Novogene, Cambridge, UK, for library preparation using primers targeting the hypervariable V4 region of the 16S ribosomal RNA gene (515F and 806R (Caporaso et al. 2011)) and sequencing on the Novaseq 6000 to generate 250 bp paired‐end reads.

### 2.3 Data Analysis

Raw sequence reads (fastq format) were denoised using DADA2 (Callahan et al. 2016) and taxonomy was assigned to amplicon sequence variants (ASVs) by applying the classify‐sklearn algorithm in QIIME 2 (v2022.2) using a Naïve Bayes classifier pre‐trained on the SILVA 138.1 database (Quast et al. 2012). The phylogenetic relationships between ASVs were determined in QIIME 2 through a multiple sequence alignment using MAFFT (Katoh and Standley 2013) and phylogenetic reconstruction using fasttree (Price, Dehal, and Arkin 2009). QIIME data artefact (qza) files were then imported into Rstudio (R Core Team 2023; v4.3.2) for subsequent analyses. These data were then converted to a *Phyloseq* object (McMurdie and Holmes 2013) and the *Decontam* package (Davis et al. 2018) was then used to identify and remove contaminant ASVs using the ‘prevalence’ method and following recommendations from (Díaz, Escobar, and Avila 2021) to identify contaminants as all sequences more prevalent in controls than true samples. The dataset was then filtered further to remove mitochondria and chloroplast sequences and retain only bacterial ASVs using the subset_taxa command in the *Phyloseq* package. Rarefaction curves were generated for all samples, with the exclusion of the negative controls, remaining after quality control and filtering using the ‘ggrare’ function in the *Ranacapa* package (Kandlikar et al. 2018), followed by rarefaction at the smallest library size (post‐filtering). The resulting rarefied counts table was then used for all subsequent analyses.

Alpha (Shannon's index) diversity was calculated using the *MicrobiotaProcess* package (Xu et al. 2023) and plotted using *ggplot2* (Wickham 2011). Statistical significance in between groups was calculated using Kruskal Wallace Rank Sum tests using the ‘kruskal. test’ function in the *stats* package v4.3.2 (R Core Team 2023) with post hoc pairwise testing using Dunn's tests with Bonferroni adjustment for pairwise testing (Dinno 2017). Differences were considered statistically significant if *p* ≤ alpha/2. Beta diversity metrics (Bray–Curtis and unweighted Unifrac) were calculated using the *Phyloseq* package with the ‘distance’ function, followed by ordination using the ‘ordinate’ function and plotting using ‘plot_ordination’. Ellipses were added to the plots using ‘stat_ellipse’ using the default 95% confidence levels assuming multivariate t‐distribution. Overall differences in beta diversity between sample types were calculated using permutational multivariate analysis of variance (PERMANOVA) with the ‘adonis2’ function in the *vegan* package (Oksanen et al. 2022), with subsequent pairwise comparisons calculated using the ‘pairwise.adonis2’ function in the *pairwiseAdonis* package (Arbizu 2017). Differences between groups were considered statistically significant if *p* ≤ 0.05. We also conducted a permutational analysis of dispersion (PERMDISP) tests using the ‘betadisperser’ and ‘permutest’ functions in the *vegan* package to assess whether observed differences in beta diversity between groups might be attributed to variations in dispersion (variance), rather than differences between group centroids. To identify whether there were statistically significant differences between samples from the different insectaries, data were subset by sample type and distance metrics were recalculated. For each sample type, ‘adonis2’ and ‘pairwise.adonis’ tests were again used to determine whether samples from the three insectaries were statistically significant, followed by ‘betadisperser’ and ‘permutest’ tests. For the larvae, larval water and adult female samples, adonis2 was also used to determine whether there were cage/tray effects by assessing the nested interaction of tray/cage within the insectary. Since ‘betadisperser’ does not support nested designs, for each of the larvae, larval water and adult female samples, the data were further subset according to the building. Distance metrics were then recalculated for each subset data and analysed using ‘betadisperser’ and ‘permutest’ tests. Relative abundance plots were created from the *Phyloseq* object, with *ggplot2*. Determination of differentially abundant bacteria between the three insectaries was carried out with the ‘ancombc2’ function in the *ANCOM* package (Lin and Peddada 2020, 2024). Multiple pairwise comparisons between each insectary were carried out using a fixed formula of insectary + sample type and controlling the overall mdFDR at 0.05 using the Holm‐Bonferroni method. Heatmaps showing the relative abundance of ASVs were generated using the ‘plot_heatmap’ function in the *Phyloseq* package.

Numbers of individuals successfully developing to pupal and adult stages in each replicate tray/cage were recorded at days two and nine respectively and visualised using *ggplot2* with differences between insectaries calculated using Kruskal–Wallis tests using the kruskal. test function in Rstudio (v4.3.2). Time to pupation was also recorded for each replicate tray and plotted. After the experiment, insectary condition measurements (temperature and relative humidity) were downloaded from the TinyTag data loggers in CSV format. Minimum, maximum and mean temperatures were calculated for each insectary and plotted in Rstudio (v4.3.2) using *ggplot*. Brown–Forsythe tests were then used to test for differences in the spread of the data between the three insectaries using the ‘bf.test’ function in the *onewaytests* package (Dag, Dolgun, and Konar 2018).

## 3 Results

### 3.1 Abiotic Environmental Factors and Mosquito Development Show Differences Between Insectaries

**FIGURE 2:**
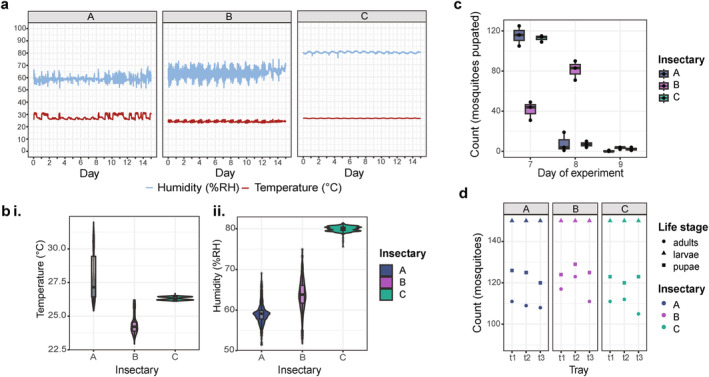
Environmental conditions and mosquito development in each insectary over the course of the experiment. (a) Temperature (°C) and humidity (% relative humidity RH) were recorded every 15 min using TinyTag data loggers in Insectaries A, B and C. Days 5/6 and 12/13 represent weekends, and there were no public holidays during this time. (b) Average and spread of recorded temperature (i) and humidity (iii) in each insectary. (c) Time taken for individuals to develop to the pupal stage in each insectary. (d) Mosquito development in each replicate tray, faceted by insectary, showing numbers of individuals successfully developed to the pupal and adult stages from an initial 150 larvae/tray.

We compared three insectaries (Figure 1a) to assess the variations in microbiomes, and putative input sources of microbes, in mosquitoes with identical host backgrounds (Figure 1b). Considering abiotic factors first, we observed clear differences in temperature and humidity. While slight differences were to be expected due to different research groups' protocols requiring slightly different set values (Table S1), we observed marked differences in the range of temperature and humidity deviations from respective set values (Figure 2a,b and Table S2). Fluctuations within each insectary correlated between temperature and relative humidity and statistically significant differences in variance between insectaries were seen for both temperature (Brown–Forsythe, *p* < 0.001, *F* (2, 2135.34) = 4842.84) and relative humidity (*p* < 0.001, *F* (2, 2909.12) = 35,479.98). Insectary A experienced the most variable temperature (average 27.81°C, standard deviation (SD) 1.78), with some days' average of 4.49°C higher than others. Insectary B, on the other hand, experienced the most variable humidity (average 51.8%, SD 3.72). Insectary C was notably more consistent than the other insectaries, with minimal temperature variations (average 26.31°C, SD = 0.12) and humidity (average 80.00%, SD = 0.60). Insectary A, the most highly used of the three, showed notable differences over the course of the experiment and Insectary B showed the most variable conditions each day and a decrease in fluctuations in the last 4 days of the experiment. We noted no major change in frequency or mode of use in any of the insectaries over the duration of our experiment except for reduced activity during weekends.

Mosquito development was monitored in the three insectaries over 14 days and showed no statistically significant difference in the numbers of mosquitoes that successfully developed to pupal and adulthood life stages in each insectary (Kruskal–Wallis, Chi‐square = 3.3504, *p* = 0.1873 and Chi‐square = 3.5862, *p* = 0.1664, respectively) (Figure 2d and Table S3). We note more variation between trays and less uniform and longer development times in Insectary B (Figure 2c) which is also the insectary with the lowest temperature, and consistently daily high fluctuations in temperature and humidity (Figure 2a,b).

### 3.2 Microbiome Complexity Varies in Mosquitoes Reared in Different Insectaries and in Their Food Sources

Altogether, 16S rRNA amplicon sequencing was carried out for 253 samples comprising 90 adult females, 90 L3 larvae, 27 larval water samples, 18 sugar solution samples, 12 tap water samples, 9 fish food samples and 7 extraction blanks (Figure 1b). After quality control and filtering, one adult female sample was removed from Insectary A and one L3 larva sample was removed from Insectary C. The extraction blanks were also then removed. These generated an average of 43,907 reads per sample (ranging from 3974 to 74,250) (Table S4). Samples were then rarefied to the lowest sampling of 3974 reads/sample, at which point the majority of rarefaction curves had plateaued (Figure S1).

**FIGURE 3:**
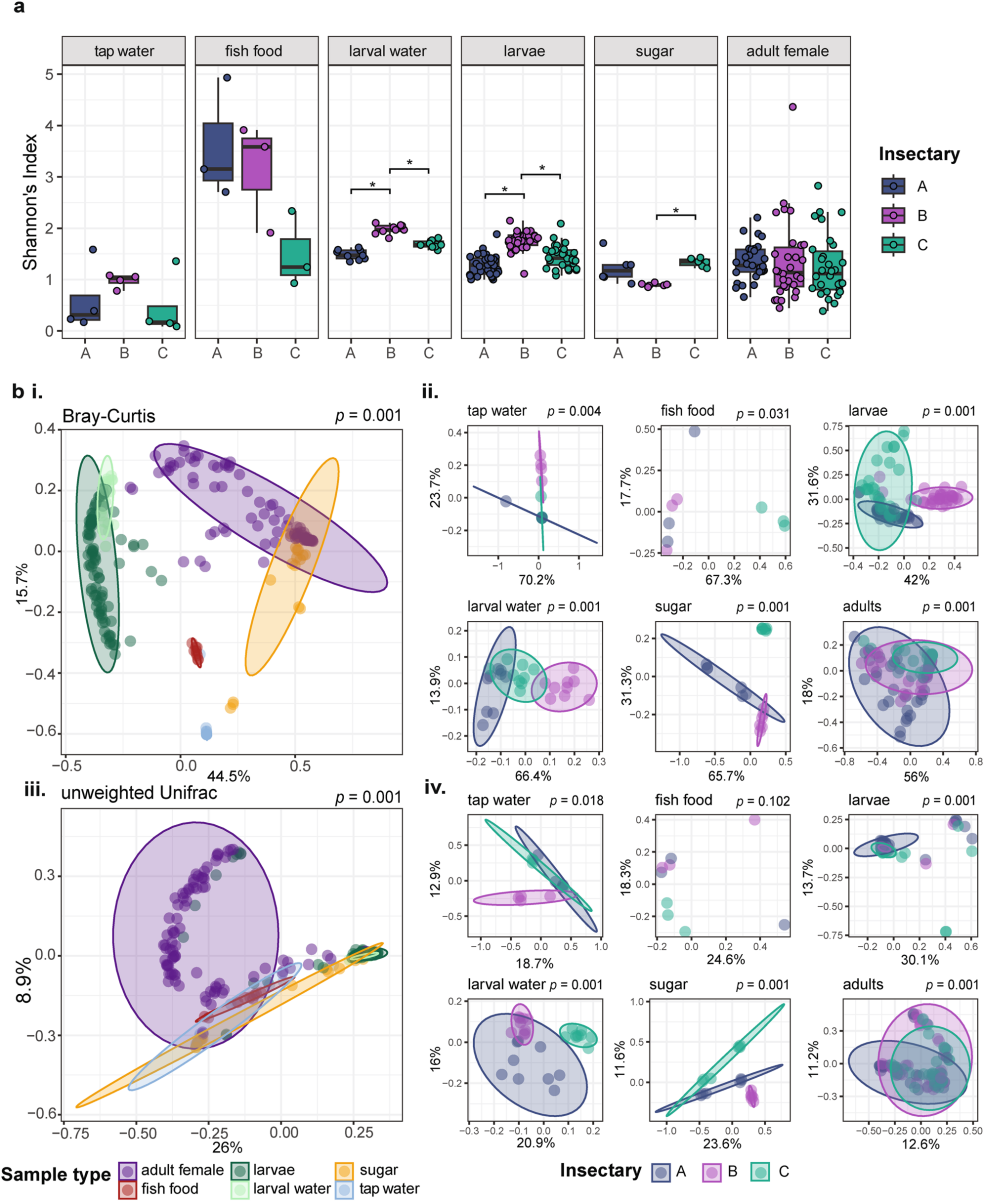
Microbial diversity amongst sample types from different insectaries. (a) Alpha diversity calculated as Shannon's index for each sample type, grouped by insectary (A, B and C). Statistically significant pairwise differences between samples from the three different insectaries, within sample types, are denoted by asterisks and are calculated using Kruskal Wallace tests with posthoc pairwise Dunn tests (*p* ≤ α/2). (b) PCoA plots showing beta diversity calculated as (i, ii) Bray–Curtis and (iii, iv) unweighted Unifrac dissimilarity metrics. Diversity was calculated using all samples passing quality thresholds, and coloured according to sample type (i, iii) Diversity metrics were then recalculated on the data subset by sample type and coloured to visualise the distribution of samples originating from each of the three insectaries (ii, iv). *p* values show results of PERMANOVA analyses to determine differences between sample types (i, iii) insectary within each sample type (ii, iv).

Overall, alpha diversity (Shannon's Index) was significantly different between sample types (Kruskal‐Wallis, Chi‐square = 65.93, *p* = < 0.001). To account for these distinct profiles per sample type, pairwise differences in alpha diversity between insectaries were compared for each sample type separately. Both larvae and larval water samples showed statistically significant pairwise differences between those from Insectary B and those from both Insectaries A (larvae: Dunn's test, *z* = −6.56, *p* = < 0.001 and larval water: *z* = −4.72, *p* = < 0.001) and C (larvae: *z* = 4.32, *p* = < 0.001 and larval water: *z* = 2.41, *p* = 0.024), with samples from Insectary B showing the highest alpha diversity (Figure 3a and Table S5). Conversely, adult mosquitoes showed no statistically significant differences in alpha diversity between insectaries. While the sugar solution samples were significantly different in alpha diversity between Insectaries B and C (*z* = 3.30, *p* = 0.002), with Insectary B exhibiting a lower diversity, there were no differences in alpha diversity of the tap water or fish food samples between any insectaries. However, the fish food samples from Insectaries A and B, which originated from the same batch, were observably more diverse than the fish food from Insectary C which originated from a different batch. These samples, comprising amongst other ingredients fish and crustacean derivatives, yeasts and algae, appeared highly variable both within and between insectaries. We do acknowledge that this dried material might also contain a substantial amount of DNA remnants from dead bacteria that were present in fish and other components.

When considering beta diversity between sample types irrespective of insectary, there were statistically significant differences using both Bray–Curtis and unweighted Unifrac distance metrics (adonis *p* ≤ 0.005 for both metrics, Figure 3b i, iii and Table S6). There were also statistically significant differences in dispersion between groups for both metrics (PERMDISP *p* ≤ 0.001 for both Bray–Curtis and unweighted Unifrac), indicating the differences in beta diversity between sample types may be partly or wholly attributed to differences in dispersion (Table S7). However, the groupings as visualised on the PCoA plots, using the Bray–Curtis metric in particular (Figure 3b i), appeared generally distinct from one another suggesting the composition of the microbiomes is an important factor in explaining the differences in beta diversity.

For each sample type, there were significant differences in beta diversity between insectaries using both metrics, except for the fish food samples using unweighted Unifrac dissimilarity (Figure 3b ii, iv and Table S8). The results of the dispersion analyses varied according to sample type and distance metric with adults, larvae and sugar solution samples showing statistically significant differences (*p* ≤ 0.05) in dispersion between insectaries using the Bray–Curtis metric, and adults and larval water with unweighted Unifrac (Table S9). Furthermore, larvae, larval water and adult female samples all showed statistically significant cage/tray effects, using both metrics (Table S8), with no statistically significant differences in dispersion between cages/trays within any of the insectaries for any sample type (Table S9).

### 3.3 Compositional Microbiome Differences in Food and at Larval Stages Converge During Mosquito Development

**FIGURE 4:**
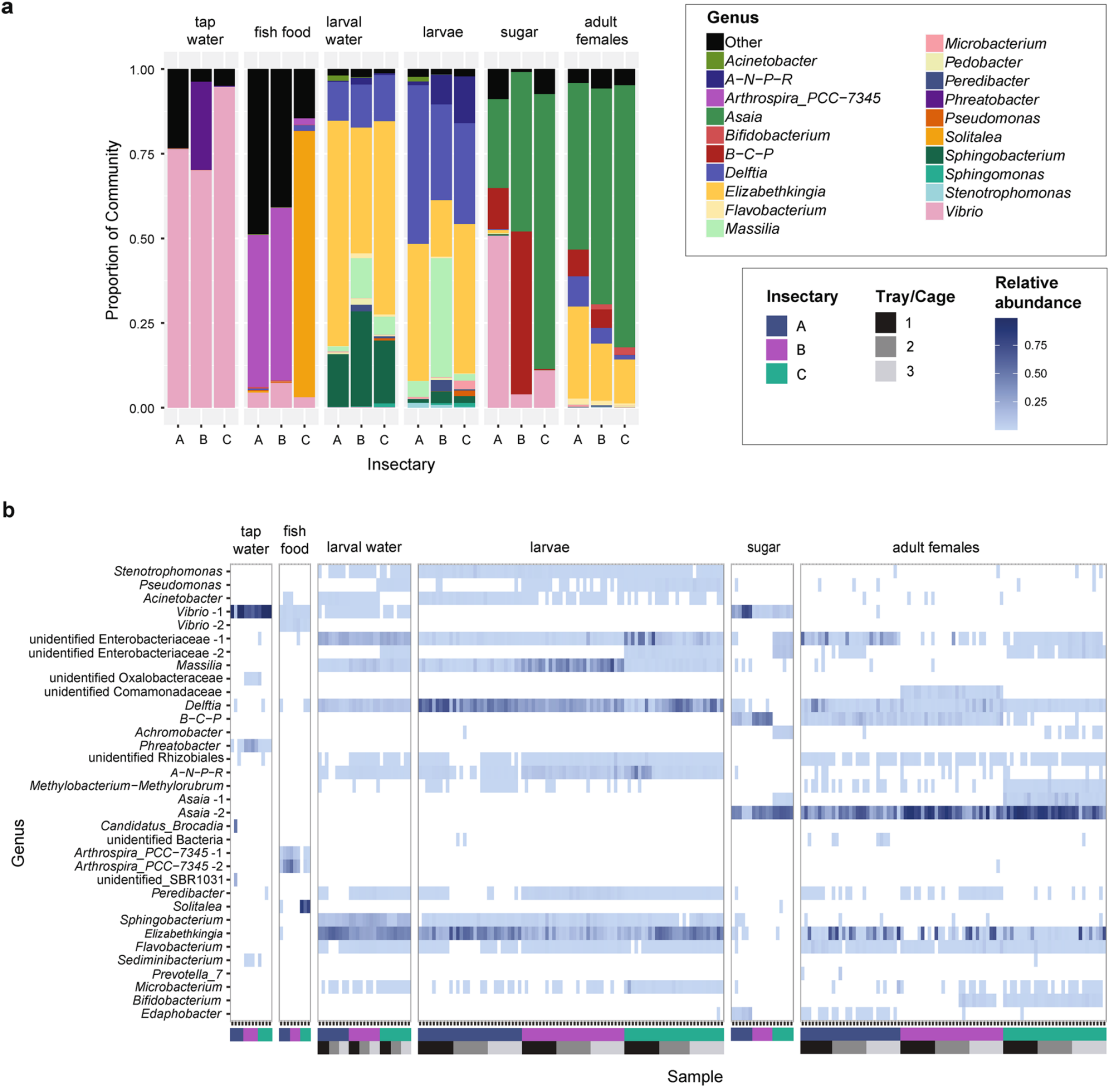
Taxonomic composition of the microbiome across sample types and insectaries. (a) Relative abundance of the top 20 most abundant genera in the data set averaged according to whether they were from Insectary A, B or C, for each sample type (tap water, fish food, larval water, larvae, sugar and adult females). All other genera were grouped together as ‘Other’. Detailed per‐sample composition is shown in Figure S2. (b) Heat map showing the relative abundance of ASVs in each sample, including all ASVs present at ≥ 5% relative abundance in at least one sample. Each row corresponds to a single ASV and is labelled on the y‐axis according to genus if known or, if unknown, the lowest taxonomic ranking known. Where there are taxonomic groups containing more than one ASV present at ≥ 5% relative abundance in at least one sample, the labels are suffixed with a number (e.g., ‘*Asaia* − 1’). Each column corresponds to a single sample, faceted by sample type. Upper colour blocks on the *x*‐axis denote the insectary of origin. Lower colour blocks denote tray/cage number within each insectary for larval water, larvae and adult female samples. Tap water, fish food and sugar samples were collected before being provided to trays/cages. Relative abundance is indicated by the blue gradient, with more highly abundant ASVs in darker shades. Zero values are indicated in white. Relative abundance values in the legend are shown as proportion data, where a relative abundance of 1 would equal 100%.

Given differences in diversity, we next assessed the taxonomic composition of the dataset for differences between different sample types and insectaries. As expected, following our observations on similarities in beta diversity, there were clear similarities in identified taxa between samples of the same sample types (Figures 4a,b and S2). Considering the composition of different sample types averaged within an insectary, adult female mosquitoes were dominated by *Asaia* and *Elizabethkingia*. Larvae and larval water samples were similar in composition and dominated by *Delftia* and *Elizabethkingia*, with *Delftia* also detected in adult mosquitoes from all insectaries, and the larval water also contained a high proportion of *Sphingobacterium* ASVs. Tap water samples were dominated by *Vibrio* and these were also present in the sugar and fish food samples albeit at lower abundances, but not present in larval or adult mosquito samples (relative abundance < 0.1%). Sugar samples from all insectaries also contained a high proportion of *Asaia* sequences. The fish food samples for all three insectaries contained dominant genera not seen in other sample types, and that varied between insectaries. The fish food from Insectary C was dominated by *Solitalea* (78.6%), which used a different fish food batch to Insectaries A and B, which were dominated by *Arthrospira*_PCC‐7345.

While the sample types contained a similar composition of main taxa in the three insectaries, the relative abundances of these genera varied by insectary (Figure 4a) and between individual samples (Figure S2). Comparing the data averaged by sample type, there were strong differences between *Massilia* (4.7%, 35.1% and 19.5%, in Insectaries A, B and C, respectively) and *Elizabethkingia* (40.5%, 16.6% and 44.1%) in the larval samples, and *Asaia* varied in sugar samples between 26.3%, 47.2% and 81.2%. Adult mosquitoes showed differences mainly in the ratio of *Asaia* (49.2%, 63.7% and 77.4%) and *Elizabethkingia* (27.2%, 16.9% and 12.9%), and a smaller but varying distribution of *Delftia* (9.0%, 4.6% and 1.3%) and *Burkholderia‐Cabelleronia‐Paraburkholderia* (7.8%, 5.5% and 0.1%). Within sample types, we observed variation between individual mosquitoes or replicate samples of non‐mosquito sample types, respectively, which appeared to be greatest in the adult females (Figure S2). We then used Ancom‐bc to identify whether there were bacteria that showed statistically significant differences between insectaries across all sample types together. Most notably, we found *Burkholderia‐Cabelleronia‐Paraburkholderia* was more abundant in Insectaries A and B than Insectary C (Ancom‐bc, log fold changes of 3.82 and 4.02, respectively, Figure S3).

Following the detection of cage/tray effects in beta diversity, we used Ancom‐bc to assess whether particular taxa were differently abundant in samples from different trays (larvae and larval water samples) and cages (adult females) within the same insectary. Tap water, sugar and fish food were not assessed as these were collected prior to providing to a tray/cage. Differentially abundant taxa were seen between trays and cages in all insectaries, however, the majority of differentially abundant taxa were specific to either particular trays or cages in just one insectary (Figure S4). Only *Delftia* was identified as differentially abundant in all three insectaries (between cages in Insectary A and trays in insectaries B and C). The differentially abundant bacteria included dominant taxa like *Massilia* which was differentially abundant between trays in Insectary C, and bacteria which were present at much lower abundances including *Stenotrophomonas* which was differentially abundant between cages in Insectary B.

To assess differential composition at higher resolution, we investigated whether different ASVs from the same genus, which can indicate different species or lineages, were present associated with insectaries and potentially restricted to specific trays/cages. While the majority of the dominant genera were only represented by one ASV, some of the dominant genera, including *Asaia* and *Vibrio*, comprised multiple ASVs, which may represent different species/lineages with different biological functions (Figure 4b). Further indicating insectary‐specific microbiomes, specific ASVs were present in different sample types from the same insectary. Most notably, one *Asaia* ASV (“Asaia 1”) was present in all adult female and sugar samples from Insectary C with average relative abundances of 4.9% and 0.6% respectively, but this was not present in samples from either of the other insectaries (Table S10). Further, one ASV within the Enterobacteriaceae (‘unclassified Enterobacteriaceae 2’) was common in abiotic samples from Insectary C (9/9 larval water samples with average relative abundance of 0.9% and 6/6 sugar samples with average relative abundance of 10.6%), and present in the majority of mosquito samples of all life stages from the same insectary (26/29 adult females with relative abundance of 1.4% and 29/29 larvae with relative abundance 0.010). However, this ASV was far less common in Insectary A, only detected in 10/29 adult females and with a lower average relative abundance of 0.2%, and it was absent from Insectary B samples.

## 4 Discussion

To understand how the insectary environment can affect microbiome composition while controlling for host background, we used a single cohort of *Ae. aegypti* eggs, split into three batches, and reared these in three different insectaries in parallel, with all work carried out by the same team across the three insectaries equally. Microbiomes can be affected by a range of external and host factors, so we measured key environmental parameters as well as assessed microbial diversity of potential input sources (tap water, fish food, larval water, sugar solution). We then recorded mosquito development and monitored the establishment of the microbiome in larvae and adult female mosquitoes.

The microbial diversity between the different insectaries was comparatively similar when considering the main taxa per sample type, except for fish food. Mosquito microbiomes were dominated by bacterial genera commonly seen in mosquito studies, including *Asaia*, *Elizabethkingia* and *Delftia* (Foo et al. 2024; Lin et al. 2021; Scolari, Casiraghi, and Bonizzoni 2019). We found differences in microbiome composition between the different insectaries which may be related to differences in abiotic and biotic factors, including bacterial input via food sources. This was particularly apparent in the adult stage, where *Asaia* was a dominant genus in both the mosquito microbiomes and the sugar solution on which they fed. One *Asaia* ASV was present in samples from all insectaries, whereas a second was present only in the sugar and adult mosquitoes from one insectary, supporting the environmental acquisition of *Asaia* from the sugar feed. While this second *Asaia* ASV was present at lower relative abundances than the first, this highlights how different bacterial inputs may be available in different insectaries and when provided with the required conditions, in this case, *Asaia* being provided with sugar solution, it may become a dominant member of the mosquito microbiome. Given that members of *Asaia* have been found to exert complex interactions with *Wolbachia* and pathogens (Hughes et al. 2014; Ilbeigi Khamseh Nejad et al. 2024; Osuna et al. 2023), this illustrates the relevance of considering potential microbial variation when conducting laboratory experiments.

Taxa observed in the input samples (tap water, fish food, larval water, sugar solution) were however only selectively present in larval and adult samples, with several dominant taxa not becoming established in the mosquito microbiomes despite representing a large proportion of the input samples. While the microbiome composition in fish food was different between insectaries, neither *Solitalea* nor *Arthrospira*, the two dominant taxa, were detected in the larvae or adult mosquito samples. Furthermore, the tap water, sugar and fish food samples all contained *Vibrio*, which however was either absent from mosquito samples or present at very low levels (< 0.1%) suggesting it is common in the insectary but is unable to successfully colonise the larvae or persist and dominate in the adult stages, potentially due to exclusionary competition via other members of the microbiome (Hegde et al. 2018). These results are supported by a previous study in which axenic mosquitoes were reared as single or mixed species groups in a common pool of larval water and through analysis of larval microbiomes suggested that mosquitoes selectively enrich for a subset of bacteria in the larval water (Hyde, Brackney, and Steven 2023). Furthermore, the physical conditions of the mosquito provide different selection pressures that favour different bacteria to those most successful in external environments, and different species and lines of mosquitoes can vary in how they control and interact with their microbiomes (Accoti et al. 2023; Muturi et al. 2016).

At the larval stage, mosquitoes varied in their microbiome diversity between the three insectaries, with individuals from Insectary B being more diverse than those from Insectaries A and C. This pattern was mirrored in the larval water, with which the mosquitoes regularly exchange microbes as they develop. This is of interest given the high variance of the conditions (temperature, humidity) in Insectary B, which might further drive a less stable microbiome. While we saw no statistically significant differences between the alpha diversity of adult mosquito microbiomes in the different insectaries, we did see specific ASV signatures associated with particular insectaries. One *Asaia* ASV was found in all adults reared in Insectary C but in none of those reared in Insectaries A or B. While we discovered *Delftia* and *Asaia* co‐occurring in the same individual adult females, previous studies indicated a potential co‐exclusion of *Delftia* and *Asaia* (da Silva, Oliveira, and Sallum 2022). However, especially given our ASV analysis demonstrated different *Asaia* ASVs in different insectaries, it remains to be determined whether this putative negative correlation is species‐ or strain‐specific and might thus differ between studies if only observed at 16S rRNA level. As 16S rRNA analysis cannot give insights into genetic determinants, it might be specific genome elements not present in all members of these genera that underpin the mechanisms responsible for causing co‐exclusion.

Additionally, one Enterobacteriaceae ASV was present in the majority of adults, larvae and larval water from Insectary C, in approximately one‐third of adults from Insectary A, but not larvae or larval water, and was absent from samples reared in Insectary B. Members of the Enterobacteriaceae can have various impacts on mosquitoes, including phenotypic effects (Dickson et al. 2017), interaction with arboviruses (Apte‐Deshpande et al. 2014; Wu et al. 2019) and other bacteria in the microbiome (Kozlova et al. 2021). Furthermore, Enterobacteriaceae exposure as larvae has been shown to influence adult phenotypes (Dickson et al. 2017). Thus, different Enterobacteriaceae might have profound impacts on subsequent experiments and our data highlights the variability even in this controlled experiment with minimal influences besides the standard rearing protocol.

The biotic and abiotic conditions also differed between the three insectaries with food sources (fish food and sugar solution) differing in microbiome composition, and environmental conditions (temperature and humidity) varying in their means and variability over time. Temperature affects diverse mosquito traits such as development, fecundity and vector competence and can affect the composition of the microbiome, including across the temperature ranges seen in our study (Mordecai et al. 2019; Onyango et al. 2020; Villena et al. 2022). The effects of humidity are less well studied, in part due to the covariance with temperature and rainfall in the field, however, it is also known to affect facets of mosquito biology such as egg production and desiccation tolerance (Brown et al. 2023). Instability in temperature and humidity, including diurnal shifts can also affect mosquitoes, including factors related to vector competence (Carrington, Armijos, Lambrechts, & Scott, 2013; Lambrechts et al. 2011; Pathak et al. 2024). Insectary C was remarkably stable in temperature and humidity compared to the other two insectaries, while the others showed a more varied pattern between and within days and larger deviations from the mean. In contrast to a previous study, we saw slower pupation times in an insectary with higher temperature fluctuations (Insectary B) (Carrington et al. 2013). Although Insectary B was also the coolest insectary, this highlights the complexity of disentangling interacting effects of means and variation in temperature, and indeed biotic factors as the larvae in Insectary B harboured the most diverse microbiomes. In addition, we observed significant differences in microbiome composition between trays and cages in all insectaries, highlighting that ideally, results should try to combine mosquitoes from multiple trays to account for this, which might be driven by position in the room (especially in relation to airflow), adjacency to other species being reared, or stochastic variation of microbes associated with individual eggs which then would get transferred into the larval water.

We appreciate not all factors could be controlled here and might have an additional impact on our results. That includes potential differences in the airflow in different insectaries and the placement of the trays and cages in relation to that which is driven practically by the spatial layout of the room. There could further be differences between cleaning regimes and disinfection methods, which we could not fully control as these are shared insectaries between multiple research groups with different experiments; a very common situation when working in research insectaries, which might impact the microbiomes. We were also not aware how the presence of other mosquito lines could impact the rearing of mosquitoes, development times or microbes present in the insectary that might get circulated in the airflow. In addition, we acknowledge the limitation of relying on 16S rRNA sequence data, which can also be derived from remnants of dead bacteria, and of only considering bacteria in the microbiome, where fungi, single‐cell eukaryotes and viruses might have further impacts (Hegde et al. 2024).

## 5 Conclusions

Laboratory experiments are commonly performed to assess diverse facets of mosquito biology under standard conditions. While factors including mosquito species and line are commonly accounted for, the microbiome can also affect experimental results and is itself influenced by diverse factors. By rearing batches of *Ae. aegypti* from a single egg cohort in three insectaries at one institution, we found insectary‐specific differences in microbiome diversity in mosquito larvae and adult females and specific ASVs associated with different insectaries and cages/trays. Our results highlight that rearing protocols, in particular, bacterial input from food sources combined with differences in the abiotic environment likely lead to compositional changes to the mosquito microbiome.

## Author Contributions

**Laura E. Brettell:** conceptualization, data curation, formal analysis, methodology, project administration, software, supervision, validation, visualization, writing – review and editing, writing – original draft. **Ananya F. Hoque:** data curation, methodology, investigation, project administration, validation, writing – original draft, writing – review and editing. **Tara S. Joseph:** data curation, visualization, validation, methodology, project administration, software, writing – original draft, writing – review and editing, investigation. **Vishaal Dhokiya:** data curation, formal analysis, investigation, methodology, project administration, software, validation, visualization, writing – review and editing. **Emily A. Hornett:** methodology, software, validation, writing – review and editing. **Grant L. Hughes:** conceptualization, funding acquisition, methodology, project administration, resources, supervision, validation, writing – review and editing. **Eva Heinz:** conceptualization, formal analysis, funding acquisition, methodology, project administration, resources, supervision, validation, visualization, writing – review and editing.

## Conflicts of Interest

The authors declare no conflicts of interest.

## Supporting information


**Figure S1.** Rarefaction curves showing a plateauing for each sample type at the rarefaction depth of 3974 reads.
**Figure S2.** Relative abundance of the top 20 most abundant genera in the data set shown for individual samples, faceted by sample type. *Allorhizobium‐Neorhizobium‐Pararhizobium‐Rhizobium* is abbreviated to *A‐N‐P‐R* and *Burkholderia‐Caballeronia‐Paraburkholderia* to *B‐C‐P*. All other genera were grouped together as ‘Other’. Upper colour blocks on the *x*‐axis denote the insectary of origin. Lower colour blocks denote tray/cage number within each insectary for larval water, larvae and adult female samples. Tap water, fish food and sugar samples were collected before being provided to trays/cages.
**Figure S3.** Heatmap showing differentially abundant bacteria between each of the three insectaries in pairwise analyses. Log fold changes are shown for each bacterial taxa, giving the highest taxonomic rank identified, which were identified as differentially abundant between insectaries (*y‐axis*) using ANCOM‐BC2. Columns denote pairwise comparisons (i.e., column one shows log fold change in Insectary B compared to Insectary A) and cell colour denotes log fold change in abundance with red representing an increase in abundance and blue a decrease. Numbers represent significant changes (adjusted *p* ≤ 0.05) and those with asterisks are significant following a further threshold of application of sensitivity analysis for pseudo‐count addition (ss filter).
**Figure S4.** Heatmap showing differentially abundant bacteria between trays and cages in the three insectaries, in pairwise analyses. Log fold changes are shown for each bacterial taxa, giving the highest taxonomic rank identified, as differentially abundant between trays (left‐hand side and cages (right‐hand side) from Insectaries A, B and C (top to bottom)) using ANCOM‐BC2. Rows show bacterial taxa and columns denote pairwise comparisons between trays (t1, t2, t3) or cages (c1, c2, c3) and cell colour denotes log fold change in abundance with red representing an increase in abundance and blue a decrease. Numbers represent significant changes (adjusted *p* ≤ 0.05) and those with asterisks are significant following a further threshold of application of sensitivity analysis for pseudo‐count addition (ss filter).
**Table S1:** Temperature, humidity and light cycle settings for the three test insectaries and average daily recorded temperature and relative humidity data using the Tinytag data logger.
**Table S2:** Raw temperature and humidity data obtained from TinyTag data loggers.
**Table S3:** Development data for each insectary showing numbers of mosquitoes developing to pupal and adult stages and duration to pupation.
**Table S4:** Sample metadata for all samples passing quality control and filtering, including the sample type, building (one experimental insectary per building was used), cage/tray as applicable, the number of reads after removal of contaminant ASVs and the accession number where the raw reads can be found on Sequence Read Archive.
**Table S5:** Result of statistical analyses of alpha diversity data using a Kruskal‐Wallis test followed by Dunn’s test for pairwise interactions. Results are presented both for the overall comparison of sample types, followed by the comparison between insectaries for each sample type separately.
**Table S6:** Results of statistical analyses of beta diversity data, using adonis2 and pairwise.adonis2 to identify statistically significant effects of sample type, insectary and sample type:insectary interactions, using both Bray‐Curtis and Unweighted Unifrac data.
**Table S7:** Results of dispersion analysis of beta diversity data (PERMDISP), comparing effects of sample type, insectary and sample type:insectary interactions, using both Bray‐Curtis and Unweighted Unifrac data.
**Table S8:** Results of statistical analyses of beta diversity data, using adonis2 and pairwise.adonis2 to identify statistically significant effects of insectary and, in the case of adult females, larvae and larval water, the nested effect of cage/tray effects within each insectary, for the different sample types, using both Bray‐Curtis and Unweighted Unifrac data.
**Table S9:** Results of dispersion analysis of beta diversity data (PERMDISP), comparing effect of insectary and, in the case of adult females, larvae and larval water, the effect of cage/tray effects within each insectary, for the different sample types, using both Bray‐Curtis and Unweighted Unifrac data.
**Table S10:** Relative abundance of ASVs in each sample, underlying the heatmap shown in Figure 4b. The data comprises all ASVs present at ≥ 5% relative abundance in at least one sample.

## Data Availability

All sequence reads are publicly available at Sequence Read Archive (SRA) under project code PRNJ1115112 and detailed accession numbers per sample are given in Table S4. All supplementary figures and tables as well as all code and underlying data files used for analysis and to generate figures are publicly available at https://github.com/laura‐brettell/insectary_comparison under zenodo id: doi.org/10.5281/zenodo.14284935.
