# Supplementary material for: Mosquitoes Reared in Nearby Insectaries at the Same Institution Have Significantly Divergent Microbiomes
Source: Environ Microbiol. 2025 Jan 8;27(1):e70027. doi: 10.1111/1462-2920.70027 (PMC11711076; doi:10.1111/1462-2920.70027)
Supplement: Supplementary file 1 — Figure S1. Rarefaction curves showing a plateauing for each sample type at the rarefaction depth of 3974 reads. Figure S2. Relative abundance of the top 20 most abundant genera in the data set shown for individual samples, faceted by sample type. Allorhizobium‐Neorhizobium‐Pararhizobium‐Rhizobium is abbreviated to A‐N‐P‐R and Burkholderia‐Caballeronia‐Paraburkholderia to B‐C‐P. All other genera were grouped together as ‘Other’. Upper colour blocks on the x‐axis denote the insectary of origin. Lower colour blocks denote tray/cage number within each insectary for larval water, larvae and adult female samples. Tap water, fish food and sugar samples were collected before being provided to trays/cages. Figure S3. Heatmap showing differentially abundant bacteria between each of the three insectaries in pairwise analyses. Log fold changes are shown for each bacterial taxa, giving the highest taxonomic rank identified, which were identified as differentially abundant between insectaries (y‐axis) using ANCOM‐BC2. Columns denote pairwise comparisons (i.e., column one shows log fold change in Insectary B compared to Insectary A) and cell colour denotes log fold change in abundance with red representing an increase in abundance and blue a decrease. Numbers represent significant changes (adjusted p ≤ 0.05) and those with asterisks are significant following a further threshold of application of sensitivity analysis for pseudo‐count addition (ss filter). Figure S4. Heatmap showing differentially abundant bacteria between trays and cages in the three insectaries, in pairwise analyses. Log fold changes are shown for each bacterial taxa, giving the highest taxonomic rank identified, as differentially abundant between trays (left‐hand side and cages (right‐hand side) from Insectaries A, B and C (top to bottom)) using ANCOM‐BC2. Rows show bacterial taxa and columns denote pairwise comparisons between trays (t1, t2, t3) or cages (c1, c2, c3) and cell colour denotes log fold change in ab [file EMI-27-e70027-s001.zip › laura-brettell/insectary_comparison-v1.1.zip!/laura-brettell-insectary_comparison-445e489/Supp_figures/Supp_fig_2.pdf]

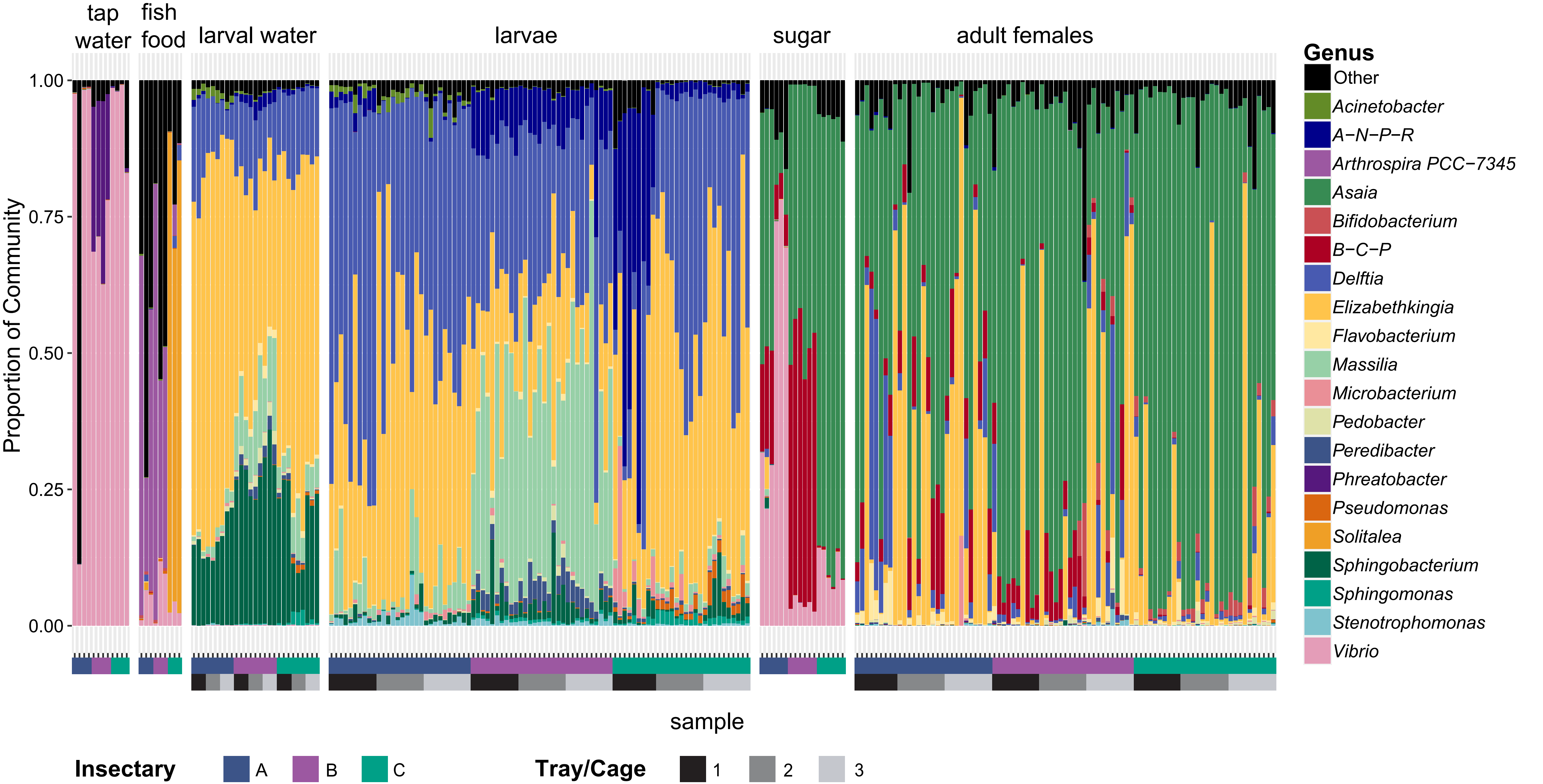

tap water
fish food
larval water
larvae
sugar
adult females
Proportion of Community
1.00
0.75
0.50
0.25
0.00
sample
Genus
Other
Acinetobacter
A−N−P−R
Arthrospira PCC−7345
Asaia
Bifidobacterium
B−C−P
Delftia
Elizabethkingia
Flavobacterium
Massilia
Microbacterium
Pedobacter
Peredibacter
Phreatobacter
Pseudomonas
Solitalea
Sphingobacterium
Sphingomonas
Stenotrophomonas
Vibrio
Insectary
A
B
C
Tray/Cage
1
2
3
